# Acromegaly Secondary to Ectopic Growth Hormone-Releasing Hormone Secretion From Metastatic Bronchial Carcinoid Tumor: A Case Report

**DOI:** 10.7759/cureus.112355

**Published:** 2026-07-09

**Authors:** Samreen F Anees, Irfaan A Abid

**Affiliations:** 1 Medicine, Idaho College of Osteopathic Medicine, Meridian, USA; 2 Metabolic and Hormonal Imbalances, Diabetes, and Endocrinology, Allina Health, St. Paul, USA

**Keywords:** acromegaly, acromegaly symptoms, ghrh, growth hormone-releasing hormone, metastatic carcinoid tumor, pegvisomant, pulmonary neuroendocrine tumor

## Abstract

Bronchial carcinoid tumors are malignant neuroendocrine tumors that typically arise from enterochromaffin cells found in the epithelial lining of the lungs.

We report a case of a 62-year-old female who was diagnosed with an atypical bronchial carcinoid tumor and subsequently underwent left pneumonectomy in December 2002. A year later, the patient developed metastases to the liver, bone, and pancreas. Over the subsequent decade, she developed clinical features of acromegaly and had persistently elevated insulin-like growth factor 1 (IGF-1) levels. Further investigation of the metastatic lesions revealed ectopic production of growth hormone-releasing hormone (GHRH).

The patient underwent multiple cycles of chemotherapy for her metastatic lesions. She also continues to require long-term therapy with octreotide (Sandostatin) and pegvisomant to control IGF-1 levels.

This case highlights the rarity of ectopic GHRH secretion from metastatic bronchial carcinoid tumors and emphasizes the importance of long-term surveillance and multidisciplinary management.

## Introduction

Bronchial carcinoid tumors are malignant neuroendocrine tumors that are well differentiated and originate from enterochromaffin cells (Kulchitsky cells) in the respiratory tract [1]. It is important to note that most neuroendocrine tumors express somatostatin receptors, allowing for a known target when considering treatment options [1].

Acromegaly is typically caused by a growth hormone-producing pituitary adenoma. Ectopic growth hormone-releasing hormone (GHRH) is an even rarer presentation, which accounts for less than 1% of all acromegaly cases [2].

We report a case of a 62-year-old female who was initially diagnosed with an atypical carcinoid tumor of the left upper lobe of the lung and subsequently developed ectopic GHRH-secreting metastatic disease involving the liver, bone, and pancreas, leading to clinical acromegaly.

## Case presentation

**Figure 1:**
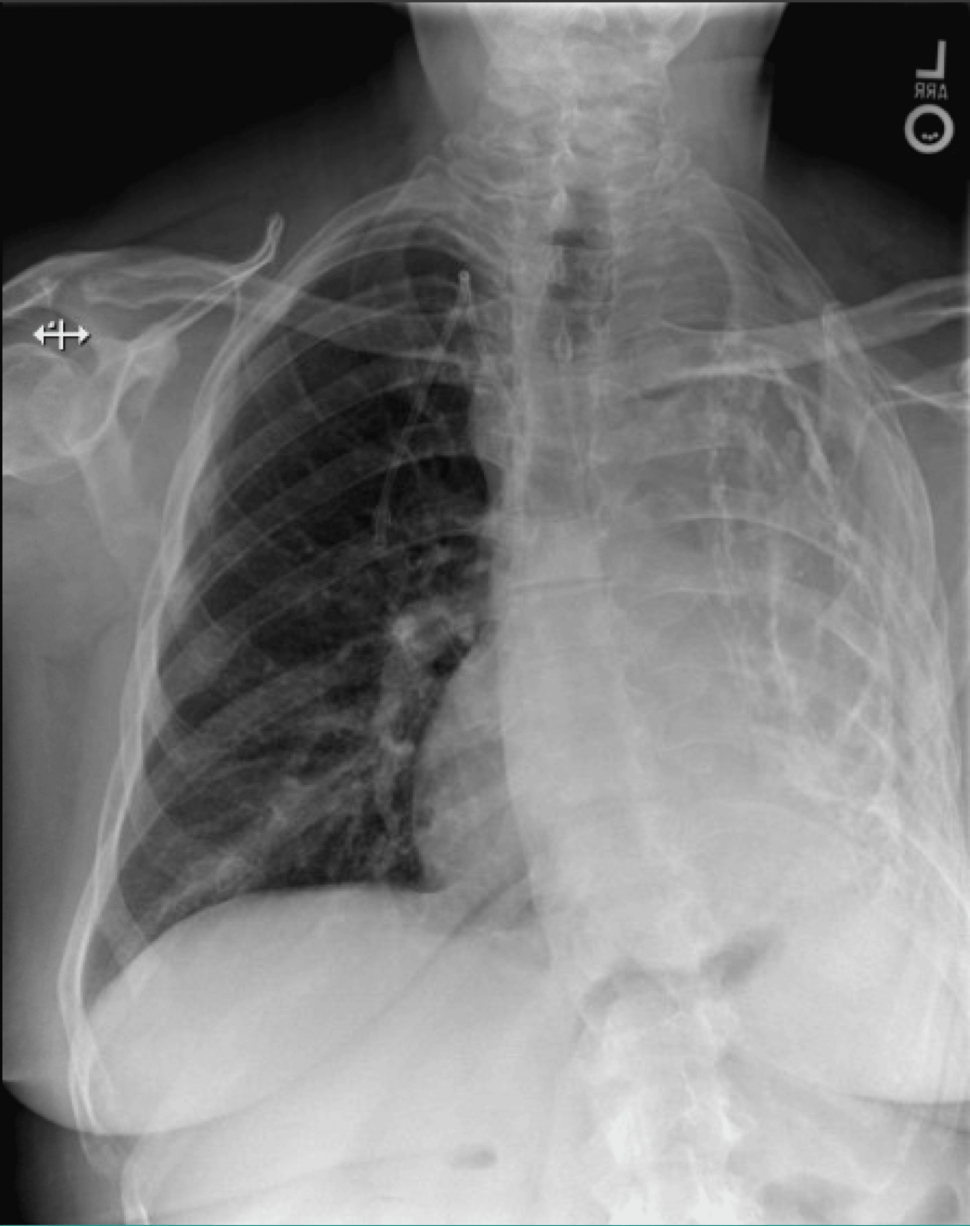
Chest radiograph depicting changes post left pneumonectomy (January 2022).

Details regarding the patient’s initial presentation are unavailable due to the age of this case. The patient was a 62-year-old female who underwent flexible fiberoptic bronchoscopy and cervical mediastinoscopy at a different institution in December 2002. This procedure revealed an atypical carcinoid tumor of the left upper lobe of the lung. Pathology identified the tumor as a neuroendocrine tumor with intrapulmonary peribronchial and tracheobronchial lymph node involvement. The final pathologic staging was T1N2, and it was removed via left pneumonectomy. A chest radiograph demonstrated postoperative changes, including complete opacification of the left hemithorax and mediastinal shift (Figure 1).

**Figure 2:**
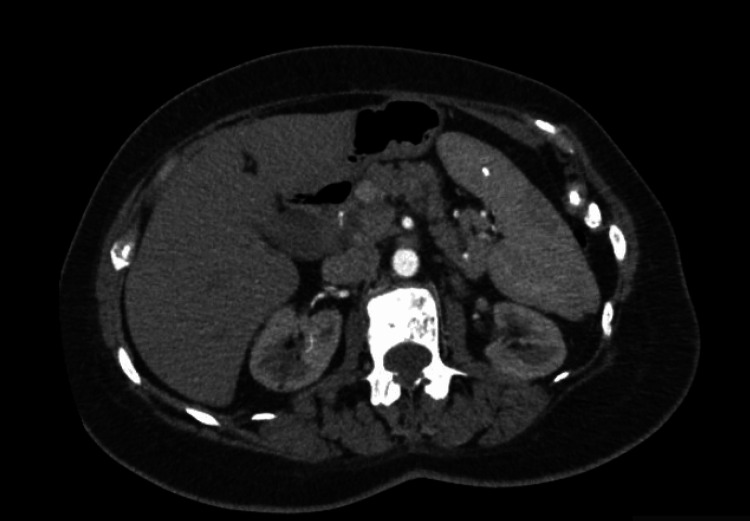
Axial computed tomography depicting metastatic involvement of the liver and pancreas (September 2021).

Computed tomography of the chest and abdomen was performed as part of routine follow-up. Imaging revealed lesions consistent with metastatic disease in the right hepatic lobe and in the head of the pancreas (Figure 2).

The patient was then started on chemotherapy and received multiple cycles of etoposide, cisplatin, R115777 (Ras inhibitor), and Tarceva. In 2010, the patient developed somatic changes consistent with acromegaly, including changes in shoe size, inability to wear her wedding ring due to hand enlargement, deepening of the voice, and difficulty chewing. She denied headaches and hyperhidrosis. Laboratory studies revealed a growth hormone level of 2.0 ng/mL (reference range: 0.01-3.61 ng/mL), an insulin-like growth factor 1 (IGF-1) level of 358 ng/mL (reference range: 37-208 ng/mL), and normal prolactin.

In March 2019, a fine-needle aspiration of the liver mass revealed a metastatic neuroendocrine tumor. Immunohistochemical studies were performed, and the cells stained positively for AE1/AE3, synaptophysin, chromogranin, and thyroid transcription factor-1 (TTF-1), supporting the diagnosis of metastatic neuroendocrine carcinoma of pulmonary origin. The histopathology and immunohistochemistry were performed at an outside institution. Thus, the original microscopic images are not available for publication. Laboratory results revealed persistently elevated IGF-1 and a GHRH level of 75 pg/mL (reference range: 5-18 pg/mL), while pituitary MRI showed no evidence of adenoma.

**Figure 3:**
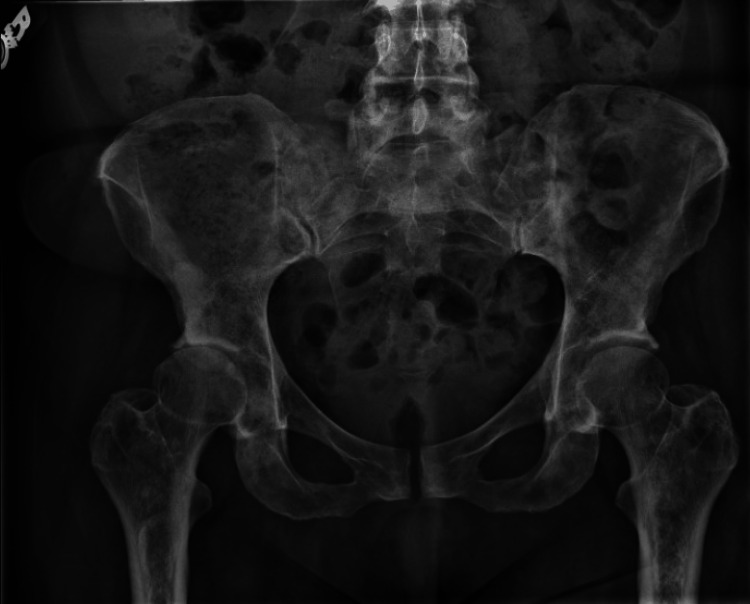
Pelvic radiograph demonstrating sclerotic osseous metastases (March 2023).

In November 2022, the patient was referred to us and started on Sandostatin. Over two years, she required dose escalation due to inadequate control of IGF-1 levels (IGF-1: 486 ng/mL). Adjunctive therapy with Dostinex, a D2 agonist, was also utilized. Improvement was noted in IGF-1 levels, as they decreased from 486 to 247 ng/mL. However, her IGF-1 levels were still considered to be out of range. In October 2024, further evaluation revealed new-onset metastasis to the thyroid gland, and the patient was started on an mTOR inhibitor known as everolimus. Repeat imaging showed stable osteoblastic lesions within the pelvis (Figure 3) and hepatic metastasis, indicating that the current pharmacotherapy regimen was effective.

Despite stable metastasis, the patient continued to exhibit inadequately controlled IGF-1 levels. Thus, the decision to discontinue Dostinex and switch to pegvisomant, a somatotropin receptor antagonist, was made in July 2025. Within two months, IGF-1 levels were 154 ng/mL (reference range: 41-279 ng/mL) and were found to be within normal limits.

## Discussion

Ectopic GHRH secretion is rare, accounting for less than 1% of all acromegaly cases [2]. As illustrated by this patient, diagnosis and management can be difficult, especially when differentiating ectopic sources from pituitary etiology. Dynamic pituitary tests, such as pituitary MRIs, were previously thought to be limited in their ability to distinguish the cause of acromegaly [3]. However, recent studies have demonstrated that certain findings may assist in providing a timely diagnosis [4].

A prior study examined T2-weighted pituitary MRI images of 30 patients with ectopic acromegaly and confirmed the presence of pituitary hyperplasia [4]. In addition, demographic differences have been noted among these patients [3]. Those with ectopic GHRH production are usually younger at the time of diagnosis (ages 36-41), while those with pituitary acromegaly are slightly older (>45 years) [3]. Moreover, ectopic GHRH production is more commonly seen in females [3]. In both cases, growth hormone (GH) and IGF-1 levels are elevated, and GH levels do not suppress even after an oral glucose load is given. However, patients with ectopic acromegaly have elevated plasma GHRH levels, suggesting that this may serve as a reliable diagnostic marker [3]. Other clinical features that may point toward ectopic acromegaly include facial flushing, dyspnea, or wheezing, peptic ulcer disease, and nephrolithiasis [3]. To our knowledge, our patient did not experience any of these symptoms prior to her initial diagnosis of an atypical carcinoid tumor.

Surgical management is critical in cases where the tumor can be localized. Complete resection of the tumor is curative; however, up to 40-50% of patients typically have metastasis at the time of diagnosis. Despite this, the prognosis is good for these patients, with a survival rate of 80% at five-year follow-up [2]. In patients with metastatic disease, optimal management has yet to be identified, but treatment with long-acting somatostatin analogues and peptide receptor radionuclide therapy has shown some benefit [2].

In cases where surgery is not curative or feasible, long-acting somatostatin analogues should be used as a first-line therapy to normalize GH levels and reduce ectopic GHRH secretion. In patients who do not respond to somatostatin analogues, pegvisomant should be added to control the progression of acromegaly and the anti-apoptotic potential of IGF-1 [5].

Regardless of the etiology, monitoring guidelines are similar for all acromegaly cases. Serial plasma GHRH values should be checked to monitor disease progression [6]. In addition, patients with acromegaly are at an increased risk of comorbidities and require systematic screening [7]. This includes a colonoscopy at the time of diagnosis due to increased risk of colonic adenomas. Echocardiography should also be performed to rule out valvular disease and cardiomyopathy. Patients should be screened for sleep apnea, hypertension, and diabetes, and managed with the appropriate treatment. Lastly, patients should undergo thyroid ultrasonography to monitor for nodular disease [7].

Patients who are receiving treatment for acromegaly require monitoring that is medication-specific. Patients taking pegvisomant for acromegaly should receive an annual pituitary MRI to evaluate pituitary size [8]. Moreover, liver function tests (LFTs) should be performed regularly during the first year of treatment, as elevation of transaminases is commonly seen in patients taking pegvisomant [9]. For patients receiving Sandostatin, evaluation of gastrointestinal side effects, gallstone formation, and changes in glycemic control should be considered [9].

The clinical course observed in this patient demonstrates the diagnostic and therapeutic challenges associated with ectopic GHRH-mediated acromegaly. This case also confirms that ectopic hormone secretion may develop years after the initial diagnosis of a neuroendocrine tumor. In addition, hormonal control was achieved in this patient after the initiation of pegvisomant, highlighting its role in patients with refractory disease. The most important takeaway is that long-term surveillance and multidisciplinary management are essential in optimizing outcomes for patients with complex metastatic neuroendocrine tumors.

## Conclusions

The clinical course observed in this patient demonstrates the diagnostic and therapeutic challenges associated with ectopic GHRH-mediated acromegaly. This case also confirms that ectopic hormone secretion may develop years after the initial diagnosis of a neuroendocrine tumor. In addition, hormonal control was achieved in this patient after the initiation of pegvisomant, highlighting its role in patients with refractory disease. The most important takeaway is that long-term surveillance and multidisciplinary management are essential in optimizing outcomes for patients with complex metastatic neuroendocrine tumors.
